# Gene-Based Pathogen Detection: Can We Use qPCR to Predict the Outcome of Diagnostic Metagenomics?

**DOI:** 10.3390/genes8110332

**Published:** 2017-11-20

**Authors:** Sandra Christine Andersen, Mette Sofie Rousing Fachmann, Kristoffer Kiil, Eva Møller Nielsen, Jeffrey Hoorfar

**Affiliations:** 1 National Food Institute, Technical University of Denmark, Kemitorvet, Building 204, DK-2800 Kgs. Lyngby, Denmark; sanan@food.dtu.dk (S.C.A.); mrsro@food.dtu.dk (M.S.R.F.); 2 Statens Serum Institut, Artillerivej 5, DK-2300 Copenhagen S, Denmark; krki@ssi.dk (K.K.); Emn@ssi.sk (E.M.N.)

**Keywords:** zoonoses, testing, feces, DNA sequencing, bioinformatics

## Abstract

In microbial food safety, molecular methods such as quantitative PCR (qPCR) and next-generation sequencing (NGS) of bacterial isolates can potentially be replaced by diagnostic shotgun metagenomics. However, the methods for pre-analytical sample preparation are often optimized for qPCR, and do not necessarily perform equally well for qPCR and sequencing. The present study investigates, through screening of methods, whether qPCR can be used as an indicator for the optimization of sample preparation for NGS-based shotgun metagenomics with a diagnostic focus. This was used on human fecal samples spiked with 10^3^ or 10^6^ colony-forming units (CFU)/g *Campylobacter jejuni*, as well as porcine fecal samples spiked with 10^3^ or 10^6^ CFU/g *Salmonella typhimurium*. DNA was extracted from the samples using variations of two widely used kits. The following quality parameters were measured: DNA concentration, qPCR, DNA fragmentation during library preparation, amount of DNA available for sequencing, amount of sequencing data, distribution of data between samples in a batch, and data insert size; none showed any correlation with the target ratio of the spiking organism detected in sequencing data. Surprisingly, diagnostic metagenomics can have better detection sensitivity than qPCR for samples spiked with 10^3^ CFU/g *C. jejuni*. The study also showed that qPCR and sequencing results may be different due to inhibition in one of the methods. In conclusion, qPCR cannot uncritically be used as an indicator for the optimization of sample preparation for diagnostic metagenomics.

## 1. Introduction

Diagnostic metagenomics is a universal, culture-independent, upcoming method with the potential to diagnose all human and veterinary infections with pathogenic microorganisms. Currently, most methods used in surveillance and diagnostics are still culture-based, including whole-genome sequencing (WGS) [1]. However, most current protocols for pre-analytical sample preparation or DNA extraction are optimized for quantitative PCR (qPCR) testing. The general objectives of sample preparation are, regardless of the final detection step, to remove assay inhibitors and produce a homogeneous sample [2]. Sample preparation and DNA extraction may also be optimized to minimize the amount of non-target DNA, e.g., from eukaryotes, and also to minimize the risk of shearing of the target DNA [3]. Pre-analytical sample preparation is especially important when analyzing fecal samples as this type of matrix is complex and has a high content of inhibitors [4].

In metagenomics, both when used for community profiling, as well as a diagnostic tool, it is important to assess the bacterial composition as accurately and close to the initial sample as possible, i.e., correct representation of both abundant and less abundant bacteria, Gram-positive and Gram-negative, as well as cultivable and non-cultivable bacteria [5]. Several studies [6,7,8,9,10] have reported that sample storage, pre-processing, DNA extraction or library preparation have a high influence on the accuracy of the bacterial composition represented. Recently, Knudsen et al. [11] concluded that it may not be possible to find one method for DNA extraction that is equally good for different fecal matrices and different pathogens.

Methods for DNA extraction are usually evaluated on their yield, concentration, and purity of DNA [2]. In most validation reports, both for qPCR and next-generation sequencing (NGS), DNA extraction is evaluated based on the DNA concentration [3,10,12,13]. However, Josefsen et al. evaluated some widely used protocols by qPCR and found no correlation between the DNA concentration and the amount of PCR-amplifiable DNA from *Campylobacter jejuni* [3]. They also showed that it was possible to obtain a higher DNA concentration from commercial kits by modifying the standard protocols. The study emphasized the need for a better method of DNA extraction, as well as improved commercial kits.

After sample preparation, DNA extraction, and sequencing, the data must be analyzed. Bioinformatics analysis in diagnostic metagenomics aims at detecting and characterizing pathogenic target microorganisms. Among possible strategies, metagenomic sequence data can be mapped against a reference genome or aligned against a database, e.g., using the Basic Local Alignment Search Tool (BLAST). Since BLAST is time consuming, another possibility is to use a metagenomics classifier, e.g., Kraken [14], MGmapper [15], CLARK [16], or MetaPhlAn [17]. A study in diagnostic metagenomics from 2017 [18] investigates the correlation between the spiking level of a bacteria and the resulting number of Kraken hits, and describes a filtering of Kraken hits to improve the specificity. The study found no linear correlation between the spiking level and the number of Kraken hits, although higher levels resulted in more hits. This is in line with the findings of McMurdie and Holmes [19], who showed that abundance in metagenomic data must be statistically modeled with a mixture distribution rather than a normal distribution.

The present study investigates, through screening of methods, whether qPCR can be used as a simple and low-cost indicator to assess the sample preparation step, before the costlier shotgun sequencing step. The study was used on human fecal samples spiked with 10^3^ or 10^6^ colony-forming units (CFU)/g of *C. jejuni*, as well as porcine fecal samples spiked with 10^3^ or 10^6^ CFU/g of *Salmonella typhimurium*. These spiking levels were chosen based on other studies [3,18] because 10^3^ CFU/g is around the lowest level detectable by the used qPCR assays and 10^6^ CFU/g can always be detected and the correlation between PCR and sequencing results can be studied quantitatively at this level. DNA was extracted from the samples using variations of two widely used kits, because different DNA extraction methods often result in different bacterial compositions and inhibitors extracted. The study evaluated these important parameters: DNA concentration, qPCR results, DNA fragmentation during library preparation, amount of DNA available for sequencing, amount of sequencing data, distribution of data between samples in a batch, and data insert size. Data were analyzed using Kraken [14] and MGmapper [15]. The novelty of the present approach, compared to what is already reported in the literature, is the direct comparison of diagnostic metagenomics and qPCR results. While qPCR is generally accepted as the gold standard in the detection of pathogens, there are no reports on qPCR as a simple indicator for optimization of sample preparation for shotgun metagenomics.

## 2. Materials and Methods

### 2.1. Sampling and Natural Presence of Campylobacter and Salmonella

Human fecal samples were taken from a presumably healthy individual with no known history of salmonellosis or campylobacteriosis, homogenized in phosphate buffered saline (PBS) 1:2 (*w/v*) for 1 min in a shaker at room temperature. The homogenate was aliquoted in 2-mL Eppendorf tubes and stored at −18 °C until use (>3 months). Porcine fecal samples were floor droppings collected in 2016 at a farm in Denmark with no known history of *Salmonella* infection. The samples were prepared according to the protocol described above for the human samples. Both fecal matrices were tested for presence of *Salmonella* and *Campylobacter* by qPCR using validated protocols (see below) after DNA extraction by QIAamp Fast DNA Stool mini kit (Qiagen, Venlo, Netherlands. Hereafter called QIAamp) standard protocol for pathogen detection.

### 2.2. Spiking

Samples were thawed at room temperature, and human samples were spiked with a liquid culture of *C. jejuni* DVI-SC181 at 10^3^ or 10^6^ CFU/g, whereas porcine samples were spiked with a liquid culture of *S. typhimurium* CCUG 31969 at 10^3^ or 10^6^ CFU/g. Spiked samples were stored at 4 °C for a maximum of three weeks until DNA extraction.

### 2.3. DNA Extraction

DNA was extracted in triplicate from both matrices by the two commercial kits Easy-DNA Kit (Thermo Fisher Scientific, Waltham, MA, USA. Hereafter called Easy-DNA) and QIAamp. For each extraction an unspiked, negative control and a process control containing sterile water were included. For QIAamp, one protocol for pathogen detection and one protocol for human DNA analysis were tested, and both were optimized as summarized in Table 1. In the standard protocols the sample size was 0.2 g and the DNA was eluted in 200 μL Buffer ATE (Qiagen) in step 14. The first two modifications, applied to both protocols, were about elution. (1) DNA eluted in 100 μL Buffer ATE; (2) DNA eluted four times. First and second time in 200 μL Buffer ATE, third and fourth time in 100 μL Buffer ATE. Eluates were pooled and concentrated in a vacuum centrifuge (Speed-Vac Concentrator, Thermo Fisher Scientific) at medium heat. DNA was dissolved in 100 μL Buffer ATE. The next two modifications, only applied to the pathogen detection protocol, were about sample size or pretreatment. (1) Fecal samples of 1 g were diluted in 9 mL 10% Chelex 100 Molecular Biology Grade Resin solution (Bio Rad, Hercules, CA, USA) in Tris-EDTA-buffer (TE-buffer). Samples were pre-centrifuged for 1 min at 1500× *g*, then the supernatant was moved to a new tube and centrifuged for 10 min at 10,000× *g*. The pellet was dissolved in 1 mL InhibitEX buffer (Qiagen) and the QIAamp protocol was followed from step 3; (2) Sample size was adjusted to 1, 5, or 10 g. Samples of 1 g were dissolved in 10 mLTE-buffer, samples of 5 and 10 g were dissolved in 50 mL TE-buffer. Samples were pre-centrifuged for 1 min at 1500× *g*, then the supernatant was moved to a new tube and centrifuged for 10 min at 10,000× *g*. The pellet was dissolved in 1 mL InhibitEX buffer and the QIAamp protocol was followed from step 3.

Sample preparation for Easy-DNA was done as follows: 1 g of feces was dissolved in 9 mL of sterile physiological saline in a 15-mL tube, vortexed for 1 min, and then centrifuged at 1500× *g* for 1 min. The supernatant was gently transferred to a new 15-mL tube and centrifuged at 10,000× *g* for 10 min. After this centrifugation, the supernatant was discarded before adding 0.5 mL PBS to the pellet and vortexing until the pellet was fully dissolved. The suspension was transferred to a new 1.5-mL tube (tube A). Tube A was centrifuged at 20,000× *g* for 5 min, after which the supernatant was decanted, and the pellet resuspended in 200 µL 1 × PBS.

DNA extraction by Easy-DNA was done as follows: 350 µL Solution A (supplied in kit) was added to suspension and vortexed for 1 s intervals until evenly dispersed. The mixture was incubated at 65 °C for 10 min. Tubes were then cooled to room temperature before adding 150 µL of Solution B (suppled in kit) and vortexing vigorously until the precipitate moved freely in the tube and the liquid was uniformly viscous. If the precipitate was hard and fixed to the tube it was loosened by knocking at the tube with a pair of scissors. Subsequently, 500 µL chloroform were added and the tube was vortexed until the viscosity decreased, and the mixture was homogeneous (between 10 s and 1 min). The tube was centrifuged at 16,000× *g* for 20 min at 4 °C to separate phases. One milliliter of 96% ethanol (stored at −20 °C) was added to a new 1.5-mL tube and kept at 4 °C. After centrifugation, 500 µL of the upper phase was transferred to the new tube with ethanol, mixed by turning the tube up and down, and incubated on ice for 30 min. Following the incubation, the tube was centrifuged at 16,000× *g* for 15 min at 4 °C and the ethanol decanted. A 500-µL 80% ethanol (stored at −20 °C) was added and the pellet was gently loosened from the tube using a pipette. The tube was centrifuged at 16,000× *g* for 5 min at 4 °C, then residual ethanol was removed with a pipette and the pellet was air-dried for 5 min. TE-RNase was prepared by mixing 100 µL TE and 2 µL 2 mg/mL RNase (supplied in kit) per tube. The pellet was re-suspended in TE-RNase and incubated at 37 °C for 1–2 h. Extracted DNA was stored at 4 °C.

### 2.4. Quantitative PCR and Measurement of DNA Concentration

DNA concentrations were measured in duplicate by Qubit high sensitivity kit (Thermo Fisher Scientific, Waltham, MA, USA). Average and standard deviation was calculated for the six spiked samples from each extraction protocol. For *Campylobacter*, the qPCR analysis was performed using a protocol validated for detection of thermotolerant *C. jejuni*, *Campylobacter coli*, and *Campylobacter lari* in chicken cloacal swabs with an infection load of 10^2^–20^3^ CFU/mL [20]. The protocol has also been applied successfully to fecal samples [3,18]. For *Salmonella*, the qPCR analysis was performed using a protocol validated for detection of *Salmonella* in meat and carcass swabs with an infection load of 1–100 CFU/25 g sample [21]. The protocol has been applied successfully on fecal samples although data is unpublished. All qPCR analyses were performed on an Mx3005P (Agilent Technologies, Santa Clara, CA, USA) and fluorescence measurements were analyzed with MxPro-Mx3005P software (Agilent Technologies). Thresholds were assigned using the default settings, i.e., standard deviation of all amplifications was determined from cycle 5 to cycle 9, and this value was multiplied by a background sigma multiplier of 10. All qPCR analyses included two non-template controls, two to three positive controls, and intern amplification control (IAC) in all wells. For all extraction protocols average Ct-values and average standard deviations were calculated for samples spiked with 10^3^ CFU/g, for samples spiked with 10^6^ CFU/g, and for IAC in all samples including negative control and process control. If no Ct was determined the value 40 was assigned, as the PCR-cycle was repeated 40 times. Ct-values were considered negative if above 40 for *Campylobacter* or above 36 for *Salmonella* according to the validation of the used qPCR assays [20,21].

### 2.5. Selection of Samples for Sequencing

The DNA concentrations from the two QIAamp standard protocols, QIAamp 1a and QIAamp 1b, were used as references in comparisons with the DNA concentration from the other QIAamp protocols and Easy-DNA. Protocols with higher DNA concentration than the standards in both matrices were selected. DNA concentrations from optimizations of QIAamp for pathogen detection were compared to those from QIAamp pathogen (1a), and similarly for the human DNA analysis protocol. The standard protocol with the highest concentration was used as a reference to compare against Easy-DNA. From the selected protocols, the subsample with the highest DNA concentration was used for sequencing.

### 2.6. Shotgun Metagenomic Sequencing and Fragmentation during Library Preparation

Library preparation was done using a Nextera XT DNA Sample Preparation kit (Illumina, San Diego, CA, USA) according to the manufacturer’s protocol. Paired-end sequencing with 2 × 250 base pair (bp) was performed on Illumina MiSeq (Illumina).

### 2.7. Measurement of Fragmentation

In the library preparation, after clean up (step 16 in Nextera XT DNA Sample Preparation Guide from January 2016, page 14), fragment sizes were measured on a BioAnalyzer (Agilent Technologies) using a high-sensitivity DNA chip and 1 μL of the library. Data were analyzed using the 2100 Expert software version B.01.03 (Agilent Technologies). The correlation area was calculated between 200 and 1000 bp.

### 2.8. Sequencing Data Analysis

Data were analyzed by Kraken [14] and MGmapper [15], where the resulting hits were evaluated at the species level. Assembled genomes from the *C. jejuni* strain and *S. typhimurium* strain used for spiking were added to the standard bacterial Kraken database to evaluate whether the method could be used for typing. The standard Kraken analysis was followed by an optimized analysis of hits to *Campylobacter* or *Salmonella* based on that by Andersen et al. [18]. Briefly, this includes scoring of hits and removal of phages and plasmids by Kraken. By BLAST, remaining hits were analyzed and reads only matching one genome were discarded, as were reads matching phages and plasmids. For MGmapper analysis, the default parameters were used, except for minimum read quality, which was adjusted to 20. The databases Bacteria, MetaHitAssembly, HumanMicrobiome, and Bacteria_draft were searched against. Comparison of Kraken hits in the different methods for DNA extraction was done in R [22] using the statistical software EdgeR (Bioconductor, Buffalo, NY, USA) [23].

### 2.9. Accession Numbers

Data were deposited in the European Nucleotide Archive (ENA) with the project numbers PRJEB21166 (human samples) and PRJEB21168 (porcine samples).

## 3. Results

### 3.1. Natural Occurrence of Campylobacter and Salmonella

Neither *Campylobacter* nor *Salmonella* were present in the human fecal samples. *Campylobacter*, but not *Salmonella*, was present in the porcine fecal samples. Therefore, human fecal samples were considered suitable for spiking with *C. jejuni*, and porcine fecal samples were considered suitable for spiking with *S. typhimurium*.

### 3.2. DNA Concentration, qPCR, and Selection of Samples for Sequencing

Average DNA concentrations and qPCR results are summarized in Table 2. DNA concentrations for human fecal samples spiked with *C. jejuni*, ranging from 0.2 to 12.9 ng/µL, were markedly lower than those for porcine fecal samples spiked with *S. typhimurium*, ranging from 0.4 to 40.5 ng/µL. There was a large variation in the standard deviations for DNA concentrations, with the higher concentrations being less accurately measured. Easy-DNA resulted in a much higher DNA concentration than QIAamp in both matrices. Compared to the QIAamp protocol with the highest yield, Easy-DNA gave a 4-fold higher yield for human feces and 2-fold higher for porcine feces. Five protocols had a higher yield than the standard protocols in both matrices: 2a, 2b, 5b, 5c, and Easy-DNA. From these five protocols the subsamples with the highest yield were sequenced. The two protocols 3b and 5a gave a higher yield than the standard in only one of the matrices; these were not sequenced.

Samples prepared by Easy-DNA and spiked with 10^6^ CFU/g *C. jejuni* were qPCR-negative, whereas all other samples spiked with 10^6^ CFU/g *C. jejuni* were qPCR-positive, with Ct-values ranging from 19.4 to 28.6. QIAamp protocols 5c and 2b had the lowest Ct-values and therefore the highest detected content of *C. jejuni*, and QIAamp protocols 3a and 4 had the highest Ct-values and the lowest detected content of *C. jejuni*. Samples from the two protocols QIAamp 4 and Easy-DNA spiked with 10^3^ CFU/g *C. jejuni* were on average qPCR-negative; samples from the remaining nine protocols were qPCR-positive, with Ct-values ranging from 28.6 to 39.1. For QIAamp protocols 2a, 2b, and 5c the Ct-values for 10^3^ CFU/g and 10^6^ CFU/g differed by approximately 10, as expected for qPCR results. The Intern Amplification Control (IAC) was markedly higher, 38.6, for Easy-DNA compared to the remaining IAC values (between 31.1 and 33.8). This indicated inhibition in the qPCR reaction, which was found to greatly influence the qPCR results of Easy-DNA. Some of the standard deviations for Ct-values were quite high, often because one or two of the three replicates were negative and assigned a value of 40, with the other replicates being positive.

Samples spiked with 10^6^ CFU/g *S. typhimurium* had Ct-values from 21.0 to 25.9, with protocols 5b, 5c, and Easy-DNA having the lowest Ct-values and 3a, 3b, and 5a having the highest. All samples spiked with 10^3^ CFU/g *S. typhimurium* were qPCR-negative. All samples had positive IAC.

### 3.3. Fragmentation, Sequencing, and Insert Size

BioAnalyzer curves were almost identical for samples prepared for sequencing in the same batch. The global maximum (hereafter referred to as “fragment size”) of all BioAnalyzer curves ranged from 563 to 1743 bp, with only five of these peaking below 1000 bp. The correlation area, i.e., the area under the curve in the range 200–1000 bp, gives an estimate of the amount of DNA available for sequencing. The correlation area was found to vary greatly between individual samples and across libraries, and ranged from 228 to 18,571.

Insert sizes were calculated from the overlap between paired forward and reverse reads. Between 22% and 85% of read pairs were overlapping by at least 12 bp, and median insert sizes ranged from 146 to 344 bp.

The amount of data from each sequencing varied greatly, with outputs from 2.57 to 15.08 Gbp (5–20 GB). Each sample in a sequencing comprised between 3% and 30% of the data. Theoretically, with seven samples in a batch, each sample should take up approximately 14% of the reads. The number of paired end reads from each sample ranged from 0.2 to 7.3 billion reads. These variations in the sequencing data available may have influenced the results.

There were no linear or ranked correlations between any two of the parameters tested: DNA concentration, qPCR, correlation area, fragment size, fraction of reads from sample, number of reads, and percent of forward and reverse reads overlapping; this was the case among all samples spiked with either *C. jejuni* or *S. typhimurium* or among samples in a batch. However, there was a correlation between the fraction of reads from the sample and the number of reads, which are always linearly correlated within a batch.

### 3.4. Data Analysis and Comparison to qPCR

The results from data analysis are compared to the qPCR results in Table 3. Target hits were evaluated at the species level, and there were many more target hits in samples spiked with *C. jejuni* compared to those spiked with *S. typhimurium*. The target ratio makes it possible to compare hits between samples with different library sizes. The highest ratio for samples spiked with *C. jejuni* was found for the protocols 1a, 5b, and 2b. The Easy-DNA sample spiked with 10^6^ CFU/g *C. jejuni* was qPCR-negative because of inhibition, but Kraken-positive. The sample from protocol 1b spiked with 10^3^ CFU/g *C. jejuni* was qPCR-negative and Kraken-positive. The sample from protocol 5c spiked with 10^3^ CFU/g *C. jejuni* was qPCR-positive with a Ct-value of 29.77 but Kraken-negative.

Interestingly, the numbers of target hits were much lower for samples spiked with *S. typhimurium* than those spiked with *C. jejuni*. The highest target ratios for samples spiked with *S. typhimurium* were found for the protocols 5c, Easy-DNA, and 5b.

Samples from the protocols 1b and 2b spiked with 10^6^ CFU/g *S. typhimurium* were qPCR-positive but Kraken-negative.

There were no linear correlations between the Kraken target ratio and the DNA concentration or qPCR at any spiking level. Furthermore, there were no linear correlations between the Kraken target ratio and any of the other measured parameters.

Statistical comparisons were performed between groups divided by spiking level, sample volume, or method for DNA extraction. Two comparisons turned out to be significant: (1) More Kraken target hits (*p* = 0.035) were found in 0.2 g samples spiked with 10^3^ CFU/g *C. jejuni* extracted by QIAamp pathogen protocols (*n* = 2) compared to QIAamp human DNA analysis protocols (*n* = 2); (2) Samples spiked with 10^6^ CFU/g *S. typhimurium* had more Kraken target hits (*p* = 0.000072) from 5 and 10 g samples (*n* = 2) compared to the 0.2 g samples (*n* = 4).

### 3.5. Analysis by MGmapper

Results from MGmapper are compared to those from Kraken in Table 3. MGmapper struggles with false positive hits in negative samples spiked with *C. jejuni*, as also seen in the raw Kraken analysis. Kraken raw and MGmapper seem to perform equally for detection of *Campylobacter*. For samples spiked with *S. typhimurium*, the MGmapper results are more similar to the final Kraken analysis.

## 4. Discussion

There are five main findings of the present study. First, qPCR and diagnostic metagenomics results were not always in agreement when the target organism was present in low concentrations or when inhibition occurred. This indicates that the qPCR results cannot be taken as an indicator of sequencing results in the optimization of sample preparation for NGS-based shotgun metagenomics. Second, diagnostic metagenomics was in some cases more sensitive than qPCR. This depended on the target organism and the chosen sequencing and qPCR protocols. Third, for 0.2-g samples spiked with 10^3^ CFU/g *C. jejuni* there were significantly more Kraken hits from samples extracted by QIAamp pathogen protocols than from those extracted by QIAamp human protocols. Fourth, for samples spiked with 10^6^ CFU/g *S. typhimurium* and extracted by QIAamp, there were significantly more Kraken hits from 5- and 10-g samples than from 0.2-g samples. Finally, none of the measured quality parameters correlated with the sequencing results.

As qPCR is often used as a reference method in diagnostic metagenomics, it is interesting that Easy-DNA samples spiked with 10^6^ CFU/g *C. jejuni* were qPCR-negative, likely due to inhibition, but Kraken-positive. Even more interesting is that the sample from the human QIAamp standard protocol (1b) spiked with 10^3^ CFU/g *C. jejuni* was qPCR-negative but not inhibited, and Kraken-positive with a target ratio similar to other samples with the same spiking level. This proves that diagnostic metagenomics can have a sensitivity equal to or higher than qPCR. Inhibition of the qPCR analysis was interpreted from the IAC Ct values, but could also have been confirmed by dilution of samples with negative IAC. Of course, the present results are influenced by the target organism, the depth of sequencing, the reference database used, and the qPCR protocol chosen. On the other hand, the sample of 10 g (QIAamp 5c) spiked with 10^3^ CFU/g *C. jejuni* was qPCR-positive, with the lowest Ct-value of all samples at that spiking level, and Kraken-negative, probably due to inhibition in the library preparation or sequencing. This was further supported by the sample of 10 g (QIAamp 5c) spiked with 10^6^ CFU/g *C. jejuni*, which was positive in both qPCR and Kraken, but with a quite low Kraken target ratio. Both 10-g samples were negative in the MGmapper analysis. The inhibition of *Campylobacter* detection in 10-g samples was contrary to the statistically significant increase in hits to *Salmonella* when increasing the sample size (QIAamp 5b and 5c). Samples extracted by the QIAamp human DNA analysis protocols (1b and 2b), spiked with 10^6^ CFU/g *S. typhimurium* were qPCR-positive but Kraken-negative. This agreed with the statistically significant fewer hits from human protocols (1b and 2b) compared to pathogen detection protocols (1a and 2a) for samples spiked with 10^3^ CFU/g *C. jejuni*.

Samples from QIAamp modifications with four eluations (3a and 3b) and with Chelex as sample preparation (QIAamp 4) were not sequenced due to the DNA concentrations being lower than those from the standard protocols. This seemed reasonable based on the literature, but may not be so, as the present study can extend the conclusion by Josefsen et al. [3] that there is no correlation between the DNA concentration and the amount of PCR-amplifiable DNA from *C. jejuni* to also include no correlation between DNA concentration and Kraken target ratio and only limited correlation between qPCR and sequencing results. In addition, Knudsen et al. [11] found no correlation between higher DNA concentration and increased community diversity or richness.

There were great variations in the DNA fragmentation, the amount of DNA available for sequencing, the amount of sequencing data, the distribution of data between samples in a batch, and the data insert size. It is surprising that there were no significant correlations between these parameters, as correlations were expected at least between the amount of DNA available for sequencing and the amount of sequencing data, and between fragmentation and data insert size. This might be due to the large variation in amount sequencing data, which is probably due to the choice and execution of library preparation and sequencing protocols. A larger study with more automated laboratory work would clarify these correlations.

In the present study data were analyzed by Kraken followed by a filtering of hits, and by MGmapper. For *Campylobacter* the raw Kraken results and the results from MGmapper were quite similar, with both having false positive hits in unspiked samples. This problem was solved for the Kraken analysis by filtering the hits to remove hits with only one reference and hits to phages and plasmids. However, this filtering makes it necessary to combine the method with a method for taxonomy-independent binning or the search for strain-specific or virulence genes to be able to type the pathogen below species level. For *Salmonella* the MGmapper results were much closer to the final, filtered Kraken results. This suggests that it is not just DNA extraction that differs between organisms, but also data analysis and the choice of software and reference database that influence the interpretation of the data. The addition of the spiking organisms to the Kraken database was done to investigate whether the method could be used for typing. However, the method cannot yet be used for typing or cannot stand alone as the target hits were often not hitting the spiking strands (data not shown). Therefore, the addition of the spiking strands was not expected to influence the results considerably.

Interestingly, the final numbers of target hits were much lower for samples spiked with *S. typhimurium* than those spiked with *C. jejuni*. This is probably because *Salmonella* is genetically more similar to other genera than *Campylobacter* is and therefore a smaller fraction of the genome is unique to *Salmonella*. However, in qPCR results great differences were also observed between *C. jejuni* spiked in human feces and *S. typhimurium* spiked in porcine feces. It is unclear whether these differences are related to organism, fecal matrix, qPCR assay, or all three. The results are, however, in agreement with Knudsen et al. [11], who concluded that a particular bacterial family will not likely be favored by a certain method of DNA isolation across different matrices.

Since none of the measured parameters were correlated with Kraken target ratio, a need to clarify which parameters are critical in the sampling, storage, sample preparation, DNA extraction, library preparation, and sequencing remains. The final conclusion of the present study is that qPCR cannot be used uncritically as an indicator of the optimization of sample preparation for NGS-based shotgun metagenomics with a diagnostic focus, as it is not granted that qPCR and sequencing results are in agreement. This conclusion is important in the design of diagnostic metagenomics studies or in pilot studies, where qPCR is often used for optimization before DNA sequencing.

## Figures and Tables

**Table 1:** QIAamp Fast DNA Stool mini kit protocol modifications.

QIAamp Protocol	Modifications to Standard Protocol
QIAamp 1a	Pathogen detection protocol, standard
QIAamp 1b	Human DNA analysis protocol, standard
QIAamp 2a	Pathogen detection protocol, eluted in 100 μL
QIAamp 2b	Human DNA analysis protocol, eluted in 100 μL
QIAamp 3a	Pathogen detection protocol, eluted 4 times
QIAamp 3b	Human DNA analysis protocol, eluted 4 times
QIAamp 4	Pathogen detection protocol, Chelex
QIAamp 5a	Pathogen detection protocol, 1 g sample
QIAamp 5b	Pathogen detection protocol, 5 g sample
QIAamp 5c	Pathogen detection protocol, 10 g sample

**Table 2:** Average DNA concentrations and quantitative PCR (qPCR) results.

DNA Extraction Method	*Campylobacter* ^a^				*Salmonella* ^b^			
	Mean DNA Conc. (ng/µL)	Mean Ct for 10^3^ CFU/g	Mean Ct for 10^6^ CFU/g	Mean IAC Ct	Mean DNA Conc. (ng/µL)	Mean Ct for 10^3^ CFU/g	Mean Ct for 10^6^ CFU/g	Mean IAC Ct
QIAamp 1a	0.2 ± 0.1	36.4 ± 1.1	24.7 ± 0.6	33.6 ± 0.6	2.3 ± 0.5	40	24.7 ± 0.3	20.5 ± 0.1
QIAamp 1b	0.5 ± 0.1	37.7 ± 1.3	24.1 ± 0.5	31.3 ± 0.3	6.8 ± 1.2	40	24.6 ± 0.5	20.4 ± 0.1
QIAamp 2a	0.5 ± 0.1	34.4 ± 0.2	23.0 ± 0.2	33.4 ± 0.7	4.6 ± 0.7	40	24.0 ± 0.2	20.4 ± 0.1
QIAamp 2b	0.9 ± 0.2	33.8 ± 0.9	21.9 ± 0.6	33.1 ± 0.6	15.3 ± 3.4	40	23.7 ± 0.1	20.3 ± 0.2
QIAamp 3a	0.2 ± 0.1	38.7 ± 1.2	27.5 ± 0.5	33.2 ± 0.5	1.7 ± 0.2	40	25.0 ± 0.3	20.4 ± 0.1
QIAamp 3b	0.9 ± 0.5	35.6 ± 0.5	23.7 ± 1.5	33.1 ± 0.5	4.3 ± 0.7	40	25.9 ± 0.2	20.4 ± 0.1
QIAamp 4	0.2 ± 0.0 ^c^	40	28.6 ± 0.1	33.8 ± 0.5	0.4 ± 0.1	40	24.2 ± 0.3	20.5 ± 0.2
QIAamp 5a	0.2 ± 0.0 ^d^	39.1 ± 1.5	25.5 ± 0.2	31.1 ± 0.4	2.4 ± 0.3	40	25.3 ± 0.5	22.4 ± 0.1
QIAamp 5b	1.3 ± 0.6	28.6 ± 6.8	24.4 ± 7.8	31.1 ± 0.3	5.6 ± 0.7	40	23.3 ± 0.2	22.4 ± 0.2
QIAamp 5c	2.8 ± 1.0	30.4 ± 0.8	19.4 ± 0.6	31.2 ± 0.2	10.4 ± 1.1	40	22.1 ± 0.3	22.3 ± 0.2
Easy-DNA	12.9 ± 1.8	40	40	38.6 ± 2.0	40.5 ± 6.6	37.3 ± 2.4	21.0 ± 0.6	26.2 ± 5.6

^a^ In human fecal samples; ^b^ In porcine fecal samples; ^c^ 0.2 ± 0.030; ^d^ 0.2 ± 0.045; IAC, intern amplification control; CFU, colony-forming units; Easy-DNA, Easy-DNA Kit.

**Table 3:** Results from qPCR and data analysis on individual samples.

DNA Extraction Method	Spiking Level (CFU/g)	qPCR Ct	Number of Reads (Millions)	MGmapper Unique Reads	Kraken Raw Target Hits	Kraken Final Target Hits	Kraken Target Ratio ^a^
*Campylobacter*							
QIAamp 1a	0	40	4.3	4222	2270	0	0.0
QIAamp 1a	10^3^	35.3	2.7	1714	1325	1	0.4
QIAamp 1a	10^6^	24.0	0.8	536	2769	1782	2235.9
QIAamp 1b	0	40	1.0	618	504	0	0.0
QIAamp 1b	10^3^	40	3.5	2541	1750	9	2.6
QIAamp 1b	10^6^	23.8	0.4	367	694	369	937.2
QIAamp 2a	10^3^	34.2	5.1	5180	2777	4	0.8
QIAamp 2a	10^6^	22.7	7.3	6711	16,367	10,344	1418.3
QIAamp 2b	10^3^	33.1	1.3	1050	823	2	1.5
QIAamp 2b	10^6^	21.3	1.0	939	2613	1656	1655.0
QIAamp 5b	10^3^	32.1	1.2	971	776	5	4.0
QIAamp 5b	10^6^	19.8	4.7	4968	17,533	9974	2131.0
QIAamp 5c	10^3^	29.8	0.5	0	236	0	0.0
QIAamp 5c	10^6^	18.8	3.2	0	612	19	6.0
EasyDNA	0	40	2.7	4201	2962	0	0.0
EasyDNA	10^3^	40	0.6	954	686	0	0.0
EasyDNA	10^6^	40	3.5	7088	6826	2021	575.6
*Salmonella*							
QIAamp 1a	0	40	0.2	0	14	0	0.0
QIAamp 1a	10^3^	40	5.2	0	315	0	0.0
QIAamp 1a	10^6^	24.5	1.5	10	687	1	0.7
QIAamp 1b	0	40	1.6	0	98	0	0.0
QIAamp 1b	10^3^	40	3.4	0	306	0	0.0
QIAamp 1b	10^6^	24.0	4.2	0	1080	0	0.0
QIAamp 2a	10^3^	40	1.7	0	115	0	0.0
QIAamp 2a	10^6^	23.8	4.7	19	963	4	0.9
QIAamp 2b	10^3^	40	6.0	0	55	0	0.0
QIAamp 2b	10^6^	23.7	0.4	0	101	0	0.0
QIAamp 5b	10^3^	40	3.5	0	201	0	0.0
QIAamp 5b	10^6^	23.3	1.8	21	1920	3	1.7
QIAamp 5c	10^3^	40	2.1	0	390	0	0.0
QIAamp 5c	10^6^	21.8	1.5	8	1885	9	6.1
EasyDNA	0	40	0.4	0	148	0	0.0
EasyDNA	10^3^	36.0	0.4	0	20	0	0.0
EasyDNA	10^6^	20.6	4.9	27	3296	14	2.9

^a^ Kraken final target hits/number of reads × 10^6^.
